# Oral treatment with T6-loaded yeast cell wall particles reduces the parasitemia in murine visceral leishmaniasis model

**DOI:** 10.1038/s41598-019-56647-w

**Published:** 2019-12-27

**Authors:** Débora B. Scariot, Hélito Volpato, Nilma S. Fernandes, Danielle Lazarin-Bidóia, Olga Borges, Maria do Céu Sousa, Fernanda A. Rosa, Andrey P. Jacomini, Sueli O. Silva, Tânia Ueda-Nakamura, Adley F. Rubira, Celso V. Nakamura

**Affiliations:** 1 Laboratório de Inovação Tecnológica no Desenvolvimento de Fármacos e Cosméticos, State University of Maringá, Maringa, 87020-900 Brazil; 2 Cellular Biology Graduate Program, State University of Maringá, Maringa, 87020-900 Brazil; 3 Faculty of Pharmacy, University of Coimbra, Coimbra, 3000-548 Portugal; 4 CNC - Center for Neurosciences and Cell Biology, University of Coimbra, Coimbra, 3000-548 Portugal; 5 Chemistry Department, State University of Maringá, Maringa, 87020-900 Brazil

**Keywords:** Drug delivery, Parasitology, Pathogens

## Abstract

Yeast cell wall particles isolated from *Saccharomyces cerevisiae* (*sc*YCWPs) have a rich constitution of β-glucan derived from the cell wall. After removing intracellular contents, β-glucan molecules are readily recognized by dectin-1 receptors, present on the cytoplasmic membrane surface of the mononuclear phagocytic cells and internalized. *Leishmania* spp. are obligate intracellular parasites; macrophages are its primary host cells. An experimental murine model of visceral leishmaniasis caused by *L. infantum* was used to evaluate the antileishmanial activity of oral administration of these particles. A low-water soluble thiophene previously studied *in vitro* against *L. infantum* was entrapped into *sc*YCWPs to direct it into the host cell, in order to circumvent the typical pharmacokinetic problems of water-insoluble compounds. We found that *sc*YCWPs + T6 reduced the parasitic burden in the liver and spleen. There was an increase in IFN-γ levels related to nitric oxide production, explaining the reduction of the *L. infantum* burden in the tissue. Histological analysis did not show signals of tissue inflammation and biochemical analysis from plasma did not indicate signals of cytotoxicity after *sc*YCWPs + T6 treatment. These findings suggested that *sc*YCWPs + T6 administered through oral route reduced the parasitic burden without causing toxic effects, satisfying requirements for development of new strategies to treat leishmaniasis.

## Introduction

In the last decade, technological advances have enabled a revolution in the treatment of medical conditions, from gene therapy and personalized medicines to 3D-printed body parts. However, when it comes to neglected tropical diseases, the advances have moved forward much more slowly. Between 2008 and 2014, only 1.5% of the novel products starting the phase-I trial were aimed at the prevention or treatment of neglected tropical diseases^1^ – among them, leishmaniasis. Despite the collaborative efforts and financial support of the World Health Organization (WHO), research institutes, pharmaceutical companies, and entrepreneurs^2,3^, the WHO estimates that up to 1 million new cases and 65,000 deaths continue to occur annually for leishmaniasis worldwide (https://www.who.int/news-room/fact-sheets/detail/leishmaniasis). The major challenges in the drug discovery and development for the treatment of leishmaniasis is not just related to the cost required for research but the biology of the parasite itself. *Leishmania* spp. are obligate intracellular parasites, which reside in effector cells of the mononuclear phagocyte system, therefore new antileishmanial molecules must be able to reach the parasites without promoting host cell damage^2,4,5^. The high toxicity and severe side-effects associated with current antileishmanial drugs often leads to treatment abandonment by patients and thus the development of drug-resistant *Leishmania* strains, highlighting a need for alternative therapies^6^.

*Leishmania* spp. can evade the host immune response by sequestering itself inside innate immune cells and inhibiting the actions of these cells that promote parasite death, such as the production of nitric oxide and pro-inflammatory cytokines. In addition, *Leishmania* spp. can induce the secretion of immunosuppressive molecules through to the late phase of the disease^7^. Therefore, restoring the regular host immune response through immunomodulatory molecules could be a potential approach to control the parasitic burden and the relapse of leishmaniasis^2,6^.

β-glucan, a polysaccharide present in the cell wall of bacteria, protists, and fungi is a well-known natural immunostimulant, used as an adjuvant in vaccines and as immunotherapy against tumors^8–11^. In Japan, β-glucan has been used to treat malignancies for decades^8,12,13^ and some studies suggest that β-glucan is also active against intracellular pathogens improving the host immune response against *Leishmania* spp., *Plasmodium* spp., *Listeria monocytogenes*, and *Toxoplasma gondii*^14,15^. β-glucan from the yeast *Saccharomyces cerevisiae* is also considered completely non-toxic and safe, and is used as an oral prebiotic and has been used in the human diet since early civilizations as a constituent of the baker’s powder and brewer’s yeast^14,15^.

The cell walls of *S. cerevisiae* consist of mannoproteins, β-glucan, and chitin, which are linked covalently, providing a stable structure that preserves the cell’s osmotic integrity and morphology^16^. A hot alkaline hydrolysis process removes the alkali-soluble content from the yeast cell wall as well as the intracellular material. This leaves the alkaline-insoluble material resulting in “ghost” or hollow β-glucan spheres with an inner cavity where it is possible to entrap active molecules^17–19^. Particles obtained from *Saccharomyces cerevisiae* yeasts can be called *sc*YCWPs (yeast cell wall particles isolated from *S. cerevisiae*) and can be used to carry the encapsulated molecules directly to target cells for the purposes of treatment^19^. *Saccharomyces bayanus, Candida utilis, Kluyveromyces fragilis, Cryptococcus curvatus, Endomyces vernalis* among others microorganisms have also been used as sources of YCWPs^20^. Currently, there is a clinical trial (phase I/II) using YCWPs in a vaccine to treat metastatic melanoma (https://clinicaltrials.gov/ct2/show/NCT02678741).

As a drug carrier, *sc*YCWPs can deliver low-water soluble molecules into cells of the mononuclear phagocyte system, such as monocytes, dendritic cells, and macrophages. *sc*YCWPs are biodegradable and biocompatible and their rich constitution of β-glucan allows an efficient and rapid recognition by macrophages and dendritic cells via receptor dectin-1-mediated endocytosis^21–23^. As a biodegradable material, *sc*YCWPs could be digested intracellularly via host-oxidative pathways, releasing the entrapped molecule and β-glucan fragments^24^. Volpato *et al*.^25^ recently showed the promising antileishmanial activity of empty *sc*YCWPs and *sc*YCWPs containing the synthetic compound thiophene (**T6**) (4-[(2*E*)-*N*′-(2,2′-bithienyl-5-methylene)hydrazinecarbonyl]-6,7-dihydro-1-phenyl-1*H*pyrazolo[3,4-*d*]pyridazin-7-one) on an *in vitro* model of *Leishmania infantum*-infected macrophages. Furthermore, studies suggest that after oral administration, *sc*YCWPs accumulate in intestinal Peyer’s patches, inside macrophages or M-cells, which move towards the *Leishmania*-infected target organs, such as the bone marrow, spleen, and lymph nodes, through the bloodstream^17,26^. Electronic micrographs in previous *in vitro* studies demonstrated that the *sc*YCWPs phagocytosed by *Leishmania*-infected macrophages can be located within the same vacuoles after the fusion between the phagosome and the parasitophorous vacuoles mediated by lysosomes action^27–30^.

Based on the findings by Volpato *et al*.^25^, the current study was proposed to investigate the efficacy of the T6-loaded immunomodulatory carrier (*sc*YCWPs + T6) in reducing the parasitic load in the visceral leishmaniasis murine model and sought to better understand how the treatment affected the *in vivo* host response. We demonstrate that oral administration of T6-loaded *sc*YCWPs reduces the splenic and hepatic parasitic burden of *L. infantum* in mice by increasing the level of IFN-γ without promoting toxic effects. Our findings also indicate an immunomodulatory effect of the *sc*YCWPs alone as a key for the antileishmanial activity of T6-loaded *sc*YCWPs.

## Results

### Treatments did not cause changes in body mass, but altered spleen mass of the mice

**Figure 1:**
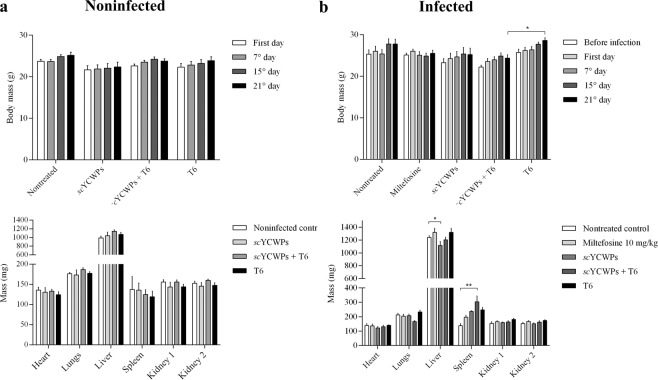
Body mass over the course of treatment and organs mass at the last day of treatment of noninfected (**a**) and *L. infantum*-infected (**b**) mice. Animals were treated with empty *sc*YCWPs (*sc*YCWPs), T6-loaded *sc*YCWPs (*sc*YCWPs + T6), **T6** alone (T6), and miltefosine was used as standard drug against visceral leishmaniasis. Results were expressed as mean ± standard error (n = 4). Significant differences at *p < 0.05 and **p < 0.01.

Neither BALB/c mice that were noninfected nor those that were infected with *L. infantum* (Fig. 1a,b) showed significant variations in body mass during 21 days of treatment compared to control groups. Between the first and last day of treatment, the body mass variation was compatible with advancing age. However, on the last day of treatment, there was a significant difference in the body mass between infected animals treated with *sc*YCWPs + T6 and **T6** alone (Fig. 1b). In terms of organ masses, the noninfected animals did not present significant differences (Fig. 1a). Compared with the untreated infected mice, the liver of animals treated with the empty *sc*YCWPs was the only organ that had lower mass among infected mice (Fig. 1b) whilst infected animals treated with *sc*YCWPs + T6 had higher spleen mass. The masses of the kidneys, lungs and hearts did not change within the infected animals, treated or not (Fig. 1b).

### T6-loaded *sc*YCWPs reduced the *L. infantum* burden in the spleen and liver

**Figure 2:**
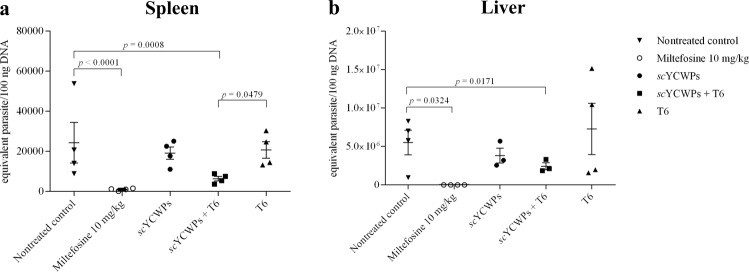
*L. infantum* burden in the spleen (**a**) and liver (**b**) determined by qPCR after treatment with empty *sc*YCWPs (*sc*YCWPs), T6-loaded *sc*YCWPs (*sc*YCWPs + T6), and **T6** alone (T6). Miltefosine was used as the antileishmanial activity control. Each point represents one animal (n = 4). At least 75% of the mice were considered in the statistical analysis. Significant differences and *p* values are represented in the graph space.

Quantitative data regarding the *L. infantum* parasitic burden in organs were obtained using the qPCR method. As expected, the parasitic burden was significantly lower in the spleen and liver after treatment with the antileishmanial drug, miltefosine. Compared to the untreated control, *sc*YCWPs + T6 caused an 85% reduction in the number of parasites in the spleen (*p* = 0.0008), and the empty *sc*YCWPs and **T6** alone did not promote a significant decrease in the number of splenic parasites (Fig. 2a). Furthermore, the T6-loaded *sc*YCWPs showed significantly greater antileishmanial activity than the **T6** alone (*p* = 0.0479), although there was no statistical difference between the empty *sc*YCWPs and *sc*YCWPs + T6. In the liver, 75% of the animals treated with *sc*YCWPs + T6 had significant reduction in the parasite burden in comparison to the untreated mice (Fig. 2b), representing a 15-fold reduction in parasite number (*p* = 0.0171). As in the spleen, empty *sc*YCWPs and **T6** alone were not able to cause a significant reduction in the hepatic parasitism in comparison to untreated control and *sc*YCWPs + T6 treated group.

### Splenic IFN-γ and TNF-α levels increased after treatment of infected mice with *sc*YCWPs and T6 loaded-*sc*YCWPs

**Figure 3:**
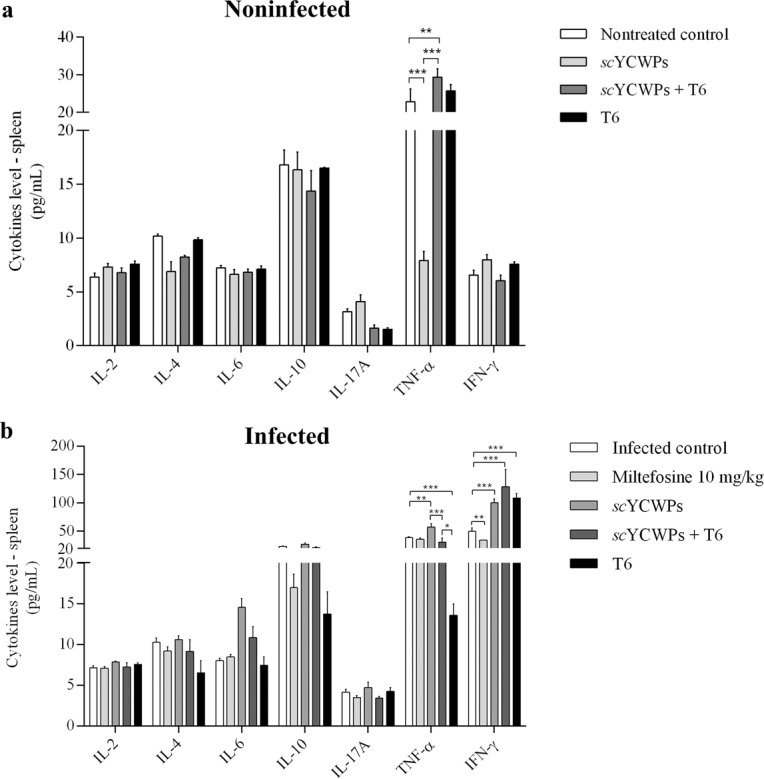
Cytokine measurements from the spleens of noninfected (**a**) and *L-infantum* infected (**b**) mice after treatment with empty *sc*YCWPs (*sc*YCWPs), T6-loaded *sc*YCWPs (*sc*YCWPs + T6) and **T6** alone (T6). Miltefosine was used as the standard drug against visceral leishmaniasis. Data were expressed as cytokine production in pg/ml and shown as mean ± standard error (n = 4). Using the BD Cytometric Bead Array (CBA) Mouse Th1/Th2/Th17 Cytokine Kit manufacturer’s instruction, 2,500 events were analyzed for each sample. Significant differences at *p < 0.05, **p < 0.01, ***p < 0.001.

To assess the immunomodulatory action of treatment, the cytokine milieu in the spleens was evaluated. In noninfected animals, the treatment with empty *sc*YCWPs caused a decrease in TNF-α compared to those that were untreated. However, the T6-loaded *sc*YCWPs were responsible for causing a significant rise in the TNF-α level compared with the untreated control and animals treated with empty *sc*YCWPs (Fig. 3a).

In infected animals, a different cytokine profile was observed, particularly in the case of IFN-γ secretion, with empty *sc*YCWPs, *sc*YCWPs + T6, and **T6** alone promoting a similar significant increment in the IFN-γ levels. With regards to the TNF-α level in the infected mice, treatment with the empty *sc*YCWPs showed a significant TNF-α increase in comparison to the untreated control. Whilst treatment with *sc*YCWPs + T6 was not significantly higher than the untreated control, the level lied between the TNF-α overproduction from empty *sc*YCWPs and the underproduction from **T6** treatment (Fig. 3b). With the exception of IFN-γ levels, treatment with miltefosine did not significantly alter cytokine levels in comparison with the untreated control. No significant variations were observed for IL-2, IL-4, IL-6, IL-10, IL-17A in all groups of infected and uninfected mice.

### Treatment with T6-loaded *sc*YCWPs does not show biochemical signs of toxicity

**Figure 4:**
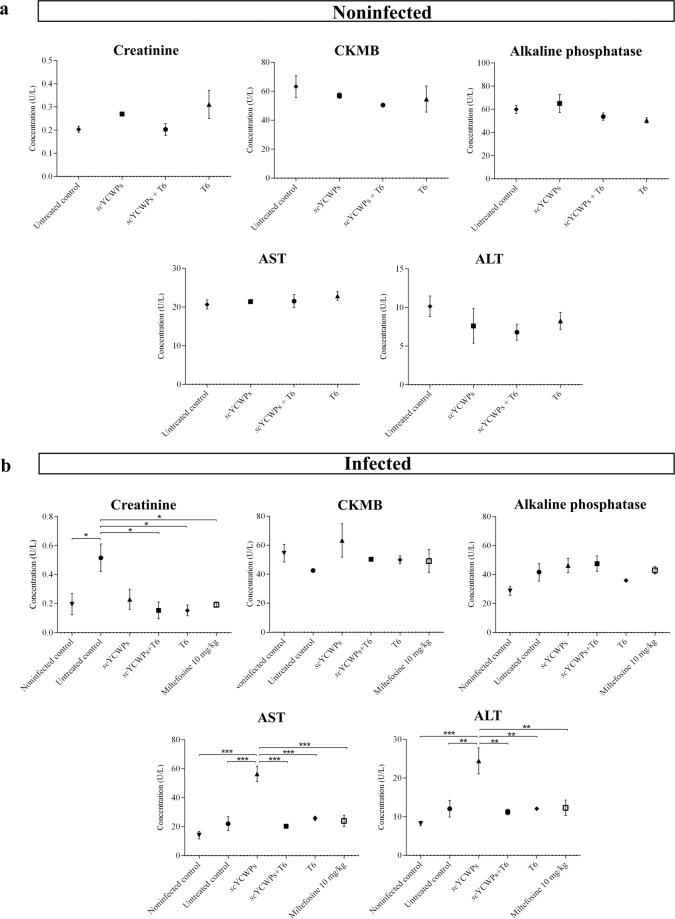
Biochemical analysis of plasma from (**a**) noninfected and (**b**) *L. infantum-*infected animals. In both assays, animals were treated with empty *sc*YCWPs (*sc*YCWPs), T6-loaded *sc*YCWPs (*sc*YCWPs + T6), and **T6** alone (T6). Miltefosine was used as the standard antileishmanial drug to treat infected mice. Renal, cardiac and hepatic functions were measured through creatinine, CKMB, and alkaline phosphatase, AST and ALT levels, respectively, by UV kinetic determination. All tests were performed in duplicate with three animals per group and the results were shown as mean ± standard error. Significant differences at *p < 0.05, **p < 0.01, ***p < 0.001.

Biochemical analyses of the plasma of noninfected animals showed the preservation of renal, hepatic and cardiac functions after treatments (Fig. 4a). The creatinine level, an indicator for renal dysfunction, was significantly higher in the untreated infected group compared to the noninfected control and the infected animals treated with *sc*YCWPs + T6, **T6** alone, and miltefosine. Biomarkers of liver damage, AST and ALT, revealed hepatic abnormalities in infected animals only after treatment with the empty *sc*YCWPs in comparison to the other groups, including the untreated mice (Fig. 4b). Treatments did not cause a significant change in cardiac function, according to biochemical analysis of CKMB.

## Discussion

The treatment of leishmaniasis has been minimally changed since the 1920s when pentavalent antimonials were first made available^31^. Severe side effects, low efficacy, development of antimony-resistant strains, and the requirement of drug administration to occur exclusively in the hospital environment are clear motivations for the discontinuation of this treatment^31^. However, while there is a clear need for therapeutic innovation, some obstacles must be overcome early in the drug discovery process. At the laboratory research level, the low water solubility of bioactive molecules impairs the *in vivo* studies by compromising pharmacokinetic parameters, including absorption, permeability and consequently bioavailability. Entrapment of these compounds in liposomes, nanoparticles, microparticles, hydrogels or microemulsions are techniques to improve the solubility, and consequently, the effectiveness of drugs^32,33^. However, the high cost of this technology could hinder the access to certain populations, particularly inhabitants of developing countries^34–37^. Liposomal amphotericin B (AmBisome) is a relevant example of this; despite a successful private-public partnership led by WHO, which has reduced the prices for AmBisome for the public sector of the eligible low-income countries, the cost of liposomal amphotericin B and hospitalization required for the parenteral drug administration are still high for the most *Leishmania* endemic countries^38–40^. Therefore, the search for new molecules or strategies to combat this disease should focus on four major requirements: enhancing leishmanicidal activity, minimizing toxicity, supporting oral administration, and being price compatible with the economic reality of the tropical endemic countries. Considering all these points, we report here the reduction of *L. infantum* burden in experimental murine visceral leishmaniasis after oral treatment with an innovative low water-soluble thiophene **T6** entrapped in immunomodulatory glucan particles (*sc*YCWPs) obtained from barker’s powder, an affordable raw material.

Spleen and liver are the most affected organs in visceral leishmaniasis, which is most severe form of the leishmaniases. Independent of the causative visceralizing *Leishmania* species, the amastigote burden in the mammalian host persists in the spleen during the entire infection period^41,42^. Thus, splenic parasite reduction is fundamental to successful therapy. In the experimental model used in this investigation, the T6-loaded *sc*YCWPs treatment reduced the *L. infantum* splenic burden. The ineffectiveness in controlling *L. infantum* burden after treatment with the empty *sc*YCWPs and **T6** alone suggests that the presence of both is fundamental for the antileishmanial activity. *sc*YCWPs may have worked as a protector structure for the antileishmanial molecule **T6**, as suggested by Ren *et al*.^43^, which demonstrated that *sc*YCWPs could avoid gastrointestinal degradation of entrapped molecules.

The hepatic infection, on the other hand, is usually self-resolving after 6–8 weeks of infection^44^. However, there is not complete elimination of parasites as their presence is essential for long-term immunity^45^. In our study, the standard drug miltefosine promoted the parasite clearance from liver almost completely and *sc*YCWPs + T6 treatment was able to cause a significant parasite burden reduction but a high number of parasites still remained. Higher doses of *sc*YCWPs + T6 or a longer treatment period could improve the hepatic *L. infantum* clearance. In the liver, amastigote-controlled growth is determined by the phagolysosome proton pump of macrophages, encoded by the SLc11a1 gene, creating Fe^2+^, Zn^2+^, Mn^2+^ deprived conditions inside phagolysosomes. These cations are essential for enzymatic processes of the parasite related to growth, virulence, and protection against host oxidative stress. A mutation in the SLc11a1 gene, resulting in pump dysfunction, is responsible for the susceptibility of the BALB/c mice to visceral leishmaniasis, raising the hepatic parasitic burden up to 100-fold^46,47^. This genetic condition may explain the high parasite number in the liver observed in this study.

*sc*YCWPs have great potential as an oral drug delivery system as they are biodegradable and biocompatible and our results show delivery to the target organs in this disease model. The rich constitution of β-glucan in the *sc*YCWPs allows an efficient and rapid recognition by macrophages and dendritic cells via receptor dectin-1-mediated endocytosis. As a biodegradable material, *sc*YCWPs can be digested intracellularly via host-oxidative pathways, releasing the entrapped molecule and β-glucan fragments. Unlike bone marrow, spleen, and lymph nodes, there is no consensus about whether the *sc*YCWPs are brought to the liver^43,48,49^, but our findings may indicate that this delivery to the liver occurs since the *L. infantum* hepatic burden presented significant reduction after oral administration of T6-loaded *sc*YCWPs.

Each *Leishmania* species and strain, which cause the spectrum of the disease, generates a dynamic profile of inflammation *in vivo* with different patterns of inflammatory mediators secretion^50^. In general, self-resolving infections are marked by a balance of Th-1 and Th-2 immune responses. An exacerbated pro-inflammatory Th-1 response, with high IFN-γ production, is typical of lesions with low healing capacity, as well as of deforming mucocutaneous wounds. Chronic and anergic leishmaniasis are characterized by an intense anti-inflammatory Th-2 response^51^. In this study there were substantial changes in the splenic IFN-γ concentrations after treatments with the empty *sc*YCWPs, *sc*YCWPs + T6, and **T6** alone. Typically, IFN-γ secretion is induced by TNF-α, which in turn induces the Th-1 response. IFN-γ is responsible for up-regulating the inducible nitric oxide synthase (iNOS) from L-arginine, releasing large amounts of nitric oxide (NO), responsible for killing intracellular parasites^52^. However, recent studies showed that high concentrations of IFN-γ were insufficient to control *Leishmania* infection in TNF-deficient mice, suggesting the need for basal TNF-α production and a NO effect^53^. It is worth noting that high NO production is considered fundamental for the resolution of leishmaniasis; however, NO production is not completely dependent on TNF-α secretion^52,54^. This could be observed in this investigation because the increase of IFN-γ levels after treatment with *sc*YCWPs + T6 was independent of an increase in TNF-α. However, TNF-α levels after treatment with **T6** were lower than the untreated control which may be related to the failure of **T6** alone to control the infection, despite the increase IFN-γ.

Some previous studies showed that the standard leishmanicidal drug miltefosine may act as an immunomodulatory molecule, stimulating IL-12 secretion and, consequently, increasing IFN-γ and NO. However, these findings depend on several parameters, such as the *Leishmania* species, animal model or type of cell, administration route, and dose considered in the studies^55^. In our model, the data suggest that miltefosine acts via an inherent effect on the protozoan, without altering cytokines level. This can be corroborated by previous findings, which revealed the effectiveness of miltefosine in immunodeficient mice and HIV-coinfected patients, indicating that its activity is independent of a T and B-cell mediated immune response^56–59^. Alternatively, miltefosine could alter the intracellular lipid metabolism of parasite, acting directly on the parasite^60^.

The absence of inflammation signals in splenic and hepatic tissue of animals treated with **T6** alone, suggests that **T6** may have reduced the inflammation and, consequently, promoted high parasite growth (Supplementary Figs. S1 and S2). These findings can reinforce the splenic tissue patterns verified by Cavancanti *et al*.^61^ in *L. infantum*-naturally infected dogs. Although hepatic granulomas precede the self-resolution of the parasitism in immunocompetent hosts, excessive inflammation may cause tissue damages and toxic symptoms^62^. Empty *sc*YCWPs did not promote a significant parasite decrease in the liver, but extensive hepatic granulomas could be observed in the histological analysis (Supplementary Fig. S2) as well as hepatic biomarkers (AST/ALT) revealed a discrete liver commitment.

In summary, T6-loaded *sc*YCWPs unite the immunomodulatory effect of the *sc*YCWPs and the active compound **T6**, creating an equilibrium between effectiveness and safety. The *sc*YCWPs + T6 treatment resulted in reduced parasite burdens in the absence of toxic signals or exacerbated inflammation in the spleen and liver of *L. infantum*-infected animals. Our findings emphasize the application of *sc*YCWPs as a drug microcarrier for oral administration. The low-cost raw material used in this study contrasts with the high cost of polymers commonly used for the development of drug delivery systems. The *sc*YCWP as a drug delivery system exhibits great potential for future drug discovery investigations against others intracellular pathogens.

## Materials and Methods

### Chemicals

*Saccharomyces cerevisiae* (baker’s yeast) was obtained from Mauripan Instant Dry Yeast. RPMI 1640 medium and fetal bovine serum were purchased from Gibco – Life Technologies Corporation. Miltefosine, poly-L-lysine, and acetone (analytical grade) were purchased from Sigma–Aldrich. PureLink Genomic DNA Mini Kit and *Leishmania* kDNA primers were obtained from Invitrogen and Integrated DNA Technologies (Coralville, IA, USA), respectively. Platinum SYBRGreen from Thermo Fisher Scientific. Permount mounting medium was purchased from Fisher Chemical and yellow eosin from InLab (São Paulo, Brazil). Histological paraffin, ethanol (analytical grade), and xylol (analytical grade) were obtained from Synth. Harris’s hematoxylin and biochemical kits for quantification of creatinine, ALT, AST, CKMB, and alkaline phosphatase were purchased from Laborclin – Bio Liquid. BD Cytometric Bead Array (CBA) Mouse Th1/Th2/Th17 Cytokine Kit was purchased from BD Bioscience (Becton, Dickinson and Company).

### Preparation and characterization of the empty *sc*YCWPs and *sc*YCWPs + T6

Empty *sc*YCWPs and *sc*YCWPs + T6 were obtained according to Volpato *et al*.^25^. Briefly, 20 g of *S. cerevisiae* powder underwent to a hot acid-base extraction using NaOH 1 M and HCl 1 M, at 85 °C. At the end of the process, the insoluble material was collected by centrifugation (2000 × g for 10 min) and washed in water, isopropanol, and acetone. The residual acetone was removed by evaporation at room temperature. This process allowed to obtain the *sc*YCWPs, rich glucan-content, by removing all intracellular structures, as well as the proteins, lipids, and mannan from the cell wall. To entrap **T6** in the *sc*YCWPs, 100 µL of **T6** solution at 500 µg/mL in acetone were added in the 10 mg of empty *sc*YCWPs, and incubated at −20 °C, for 2 h. Next, the acetone was removed by evaporation at room temperature. This sequence was repeated 5 times to promote the **T6** accumulation inside *sc*YCWPs. The entrapment efficiency was determined by quantifying of the not entrapped **T6** on spectrophotometer (Shimadzu UV-1700 PharmaSpec), at 382 nm. *sc*YCWPs and *sc*YCWPs + T6 were characterized by Volpato *et al*., through of transmission electron microscopy, and zeta potential, Fourier transform infrared (FT-IR), and X-ray diffraction analysis^25^.

### Infective parasite maintenance

*Leishmania infantum* MCAN/BR/97/P142 (syn. *L. chagasi*) promastigotes in a stationary phase of growth (7–8 days) were inoculated intraperitoneally in a female BALB/c mouse. After 30 days, the spleen was removed and maintained in RPMI 1640 medium over 15 days, when infective metacyclic promastigote forms were detected in the culture supernatant. These infective parasites were maintained in RPMI 1640 medium supplemented with 10% of fetal bovine serum (FBS). After the first passage, the parasites were in the stationary phase by the eighth day.

### Visceral leishmaniasis murine model

Female BALB/c mice (7–8 weeks old) were infected with *L. infantum* promastigotes at the stationary phase of growth, obtained as described above. A suspension containing 1 × 10^8^ promastigotes/mL in 0.01 M PBS was inoculated intraperitoneally. Each group of mice was kept in a separate cage (four animals per cage), at room temperature (25 ± 1 °C), and at a 12-hour light-dark cycle. Animals received water and food *ad libitum*. At the end of 30 days post-infection, treatment was started and lasted 21 days. The infected animals were divided into 5 groups: non-treated animals; treated with 10 mg of miltefosine/kg/day; treated with 3 mg of empty *sc*YCWPs/mouse; treated with 3 mg of *sc*YCWPs/mouse containing 45.7 µg of **T6** (or 15.23 µg of **T6**/mg *sc*YCWPs); and treated with 45.7 µg of free **T6**/mouse (equivalent to that containing in 3 mg of *sc*YCWPs + T6). The mass of **T6** contained in the *sc*YCWPs was determined in previous study by Volpato *et al*., according to the encapsulation efficiency data^25^. All tested treatments used water as the vehicle. Noninfected animals were used as negative controls. The mice were weighed once a week. On the last day of the treatment, the animals were euthanized using a lethal dose of isoflurane and oxygen. Blood was collected by cardiac puncture and, after centrifugation, plasma was isolated and stored. Heart, lungs, spleen, liver and kidneys were collected, weighed and stored for further analysis. In an additional assay, female BALB/c mice (7–8 weeks old) were maintained healthy and received the same treatment as the infected mice (except for miltefosine treatment), in order to evaluate the basal response caused by the treatments.

### Ethics statement

All animal experiments were approved by the Ethical Committee on the Use of Animals (CEUA) from State University of Maringá (Protocol number 1323011116), following the requirements provided by the Regulatory Resolutions numbers 33/2016 and 39/2018 of the Ministry of Science, Technology, Innovations and Communications of Brazil.

### Quantitative real-time PCR

Spleen and liver were used to quantify the parasitic load. DNA was extracted from the tissues using the PureLink Genomic DNA Mini Kit, according to the manufacturer’s instructions. Briefly, about 10 and 25 mg of spleen and liver, respectively, were weighed and mixed with digestion buffer and proteinase K. After 4 h in a hot water bath, the samples were centrifuged at maximum rotation, adding RNase A and lysis buffer. DNA was precipitated in ethanol, purified in spin columns, washed and eluted in elution buffer. Real-time PCR was performed using the LightCycler 480 system (Roche Diagnostics Brussels, Belgium) according to the manufacturer’s instructions. Reactions were performed in a 20 µL final volume with 300 nM of *Leishmania* kDNA specific forward primer 5′-CTTTTCTGGTCCTCCGGGTAGG-3′ and reverse primer *5*′*-*CCACCCGGCCCTATTTTACACCAA-3′, previously described^63^ and Platinum SYBRGreen qPCR. Each PCR reaction contained 100 ng genomic tissue DNA. The amplification protocol consisted of a denaturation phase at 95 °C for 10 s, then 35 cycles of amplification 95 °C for 15 s, 60 °C for 60 s. At the end of each run, melting curve analysis was performed from 65 °C to 95 °C to monitor primer dimers or non-specific product formation. Each LightCycler run contained two negative controls (no DNA added to the reaction), and each DNA sample was quantified in duplicate. A standard curve was established using purified *L. infantum* DNA; serial dilutions, ranging from 100 to 0.001 ng of DNA parasites, were introduced into reaction tubes in triplicate. The standard curve was generated by LightCycler96 software and used to calculate the equivalent parasite in each sample. The equivalent parasites were calculated considering 200 fg of DNA/*Leishmania* spp.^63–65^. The data from four animals per group were considered to express the results of qPCR.

### Histological evaluations

Spleen and liver were collected, fixed in Bouin’s solution, maintained in ethanol 70%, and processed for histological analysis by paraffin inclusion. Briefly, the organs were serially dehydrated in ethanol (80%, 90%, and 100%) and finally in xylol for at least 12 h. Paraffin substituted the xylol, gradually, at 70 °C, until complete inclusion. The paraffin blocks were cut using a microtome (Leica Biosystems), at 4 µm thickness. The sections were collected on two slides previously prepared with poly-L-lysine, stained in Harris’ hematoxylin-yellow eosin, and mounted using Permount mounting medium. Spleen and liver from four animals were processed and analyzed. Histological analysis from spleen, bone marrow and lymph nodes are the gold standards for identifying *Leishmania* spp. via observation of intracellular amastigotes. Nevertheless, because of the high sensitivity and the ability to quantify parasite burden, quantitative real-time PCR has been considered the preferred technique for diagnosis. For this reason, parasite quantification was performed by qPCR in this study, and histological analysis evaluated just the general characteristics of the tissues, with qualitative purposes.

### Cytokine measurement

Contrary to the hepatic parasitic load, *Leishmania* infection is not typically self-resolving in the spleen. Thus, cytokine level measurements were carried out only on the spleen samples. For cytokine quantification, a BD Cytometric Bead Array (CBA) Mouse Th1/Th2/Th17 Cytokine Kit was used. First, the BD FACSCalibur flow cytometer was calibrated according to the manufacturer’s instructions. To obtain a standard curve, the mouse Th1/Th2/Th17 Cytokines Standard, provided by the kit, was reconstituted and serial dilutions were prepared, followed by the addition of the Mix of Capture Beads and the Mouse Th1/Th2/Th17 PE Detection Reagent, and incubated for 2 h in the dark at room temperature. Next, the samples were prepared; the previously-weighed spleen was immersed in 0.01 M PBS at 100 µg spleen/mL. The organ was macerated using ultrasonic equipment (Hielscher UP400St, maximum potency, 10 s) and placed in a cold-water bath to avoid heat degradation of the cytokines. The macerated organ was centrifuged and prepared according to the manufacturer’s instructions. Briefly, 50 µL of the samples were mixed with 50 µL of the Mix of Capture Beads and 50 µL of the Mouse Th1/Th2/Th17 PE Detection Reagent, then incubated for 2 h in the dark at room temperature. Subsequently, the samples were washed and resuspended in Wash Buffer provided by the kit, and the samples were analyzed on the BD FACSCalibur cytometer, using FL-3 as the main parameter. For the negative control, a singlet population in the FSC *vs* SSC dot plot was determined as an R1 region gate. To analyze the samples, the number of events was set to 2,100 within the R1 region gate, ensuring approximately 300 events per Capture Bead, according to the manufacturer’s instructions. The data from four animals per group were necessary to quantify the cytokines level using the FCAP Array Software 1.01.

### Biochemical analysis

Renal, hepatic and cardiac functions were evaluated from infected and uninfected animals by measuring the creatinine, alanine aminotransferase (ALT), aspartate aminotransferase (AST) and alkaline phosphatase, and creatine kinase MB (CKMB), respectively, using kits for kinetic UV determination. Non-hemolyzed plasma was prepared according to the manufacturer’s instructions. The reaction was measured on a Flexstation Microplate Reader at a controlled temperature. From each animal, two reactions were prepared. The reduced volume of blood and plasma obtained from mice allowed all tests to be performed in duplicate from three animals per group.

### Statistical analysis

Numerical results from each group were expressed as the average ± standard error (SE). Statistical analysis was performed with GraphPad Prism software, and the statistically significant difference was determined by the one-way ANOVA test. For cytokine measurements, two-way ANOVA tests were used to examine data with multiples variables, and Tukey’s post-test was used to evaluate the intergroup differences. P values less than 0.05 were considered statistically significant.

## Supplementary information

Supplementary Information

## Data Availability

The datasets analyzed during the current study are available from the corresponding author on reasonable request.
