# Supplementary material for: Oral treatment with T6-loaded yeast cell wall particles reduces the parasitemia in murine visceral leishmaniasis model
Source: Sci Rep. 2019 Dec 27;9:20080. doi: 10.1038/s41598-019-56647-w (PMC6934808; doi:10.1038/s41598-019-56647-w)

**Oral treatment with T6-loaded yeast cell wall particles reduces the parasitemia in murine visceral leishmaniasis model**

Débora B Scariot^1+^, Hélito Volpato^1+^, Nilma S Fernandes^2^, Danielle Lazarin-Bidóia^1^, Olga Borges^3,4^, Maria do Céu Sousa^3,4^, Fernanda A Rosa^5^, Andrey P Jacomini^5^, Sueli O Silva^1^, Tânia Ueda-Nakamura^1^, Adley F Rubira^5^, Celso V Nakamura^1*^

^*^ cvnakamura@gmail.com

^+^ these authors contributed equally to this work

^1^ Laboratório de Inovação Tecnológica no Desenvolvimento de Fármacos e Cosméticos, State University of Maringá, Maringa, 87020-900, Brazil

^2^ Cellular Biology Graduate Program, State University of Maringá, Maringa, 87020-900, Brazil

^3^ Faculty of Pharmacy, University of Coimbra, Coimbra, 3000-548, Portugal

^4^ CNC - Center for Neurosciences and Cell Biology, University of Coimbra, Coimbra, 3000-548, Portugal

^5^ Chemistry Department, State University of Maringá, Maringa, 87020-900, Brazil

**Supplementary Information**

**Histological analysis revealed signs of inflammation in the liver and spleen after treatment with empty *sc*YCWPs**

Noninfected animals had standard spleen architecture, with distinct white and red pulp (Fig. S1 – a and b). In the spleens of infected animals, high numbers of amastigotes within macrophages was observed. The macrophages had large amounts of cytoplasm, and the persistence of plasma cells, a sign of tissue inflammation, could also be observed (Fig. S1 – c and d). Miltefosine did not appear to cause changes in spleen architecture, and few amastigotes were detected (Fig. S1 – e and f). After treatment with the empty *sc*YCWPs (Fig. S1 – g and h) there was extensive presence of plasma cells and the number of intracellular amastigotes remained high. Large infected macrophages could also be observed after *sc*YCWPs + T6 treatment, without signs of inflammation (Fig. S1 – i and j). Treatment with **T6** alone promoted moderate-to-extensive disorganization of white pulp, losing the distinction of the typical regions, with remarkable presence of amastigotes (Fig. S1 – k and l).

In the liver of noninfected animals, the standard structure of hepatocytes, sinusoids and Kupffer cells could be observed (Fig. S2 – a and b). Hepatocytes revealed a high number of vesicles and granules, consistent with their secretory role. Infected animals showed amastigotes inside the Kupffer cells, in addition to many granulomas composed primarily of lymphocytes and infected macrophages (Fig. S2 – c and d). No amastigotes were found in the liver after treatment with miltefosine, and Kupffer cells showed discrete swelling (Fig. S2 – e and f). Livers from animals treated with the empty *sc*YCWPs showed signs of inflammation characterized by granulomatous lesions around blood vessels, involving primarily Kupffer cells and plasma cells (Fig. S2 – g and h). Large Kupfer cells could be observed after *sc*YCWPs + T6 treatment (Fig. S2 – i and j). These same signs were rarely found in the livers of animals treated with **T6** alone (Fig. S2 – k and l).

**Supplementary Figure S1 –** **Histological analysis of the splenic tissue.** (a and b) noninfected mouse; (c and d) infected mouse; (e and f) mouse treated with miltefosine 10 mg/kg/day; (g and h) mouse treated with empty YCWP; (i and j) mouse treated with YCWP + T6; (k and l) mouse treated with T6. Details show large macrophages (head arrows) and amastigotes (arrows). Asterisks (*) indicate the splenic granulomas; (wp) white pulp; (rp) red pulp; (v) blood vessel. The spleen sections from four animals per group was collected separately in duplicate. Hematoxylin-eosin staining.

**
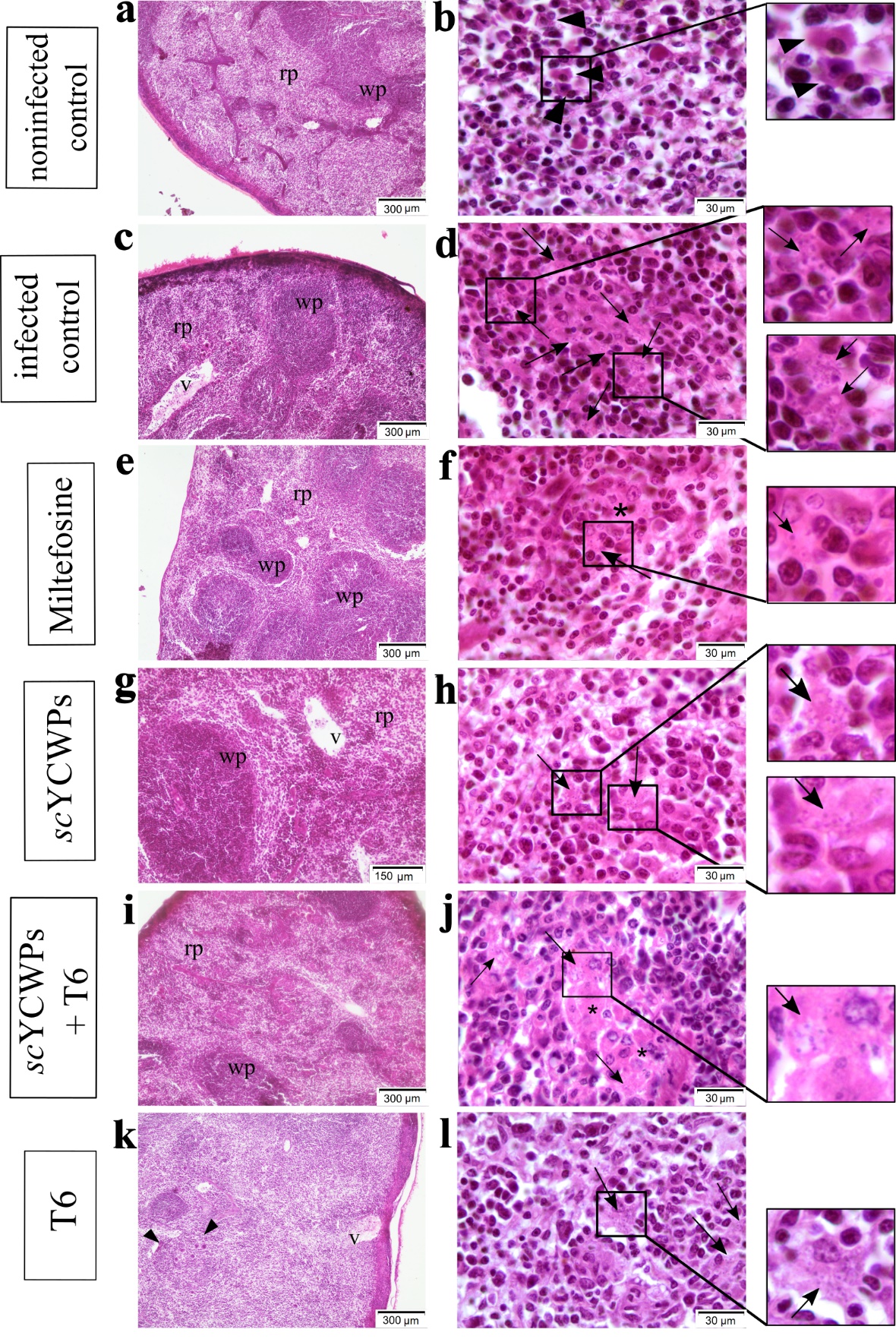
**

**Supplementary Figure S2 – Histological analysis of the hepatic tissue**. (a and b) noninfected mouse; (c and d) infected mouse; (e and f) mouse treated with miltefosine 10 mg/kg/day; (g and h) mouse treated with empty YCWP; (i and j) mouse treated with YCWP + T6; (k and l) mouse treated with T6. Black arrows indicate the presence of inflammatory infiltration. Head arrows indicate the of Kupffer’s cells. Amastigotes can be seen in the b and j (yellow arrows). Asterisks (*) indicate the hepatic granulomas. Details show granulomas (*) and amastigotes (green arrows). (e) erythrocytes. The liver sections from four animals per group was collected separately in duplicate. Hematoxylin-eosin staining.


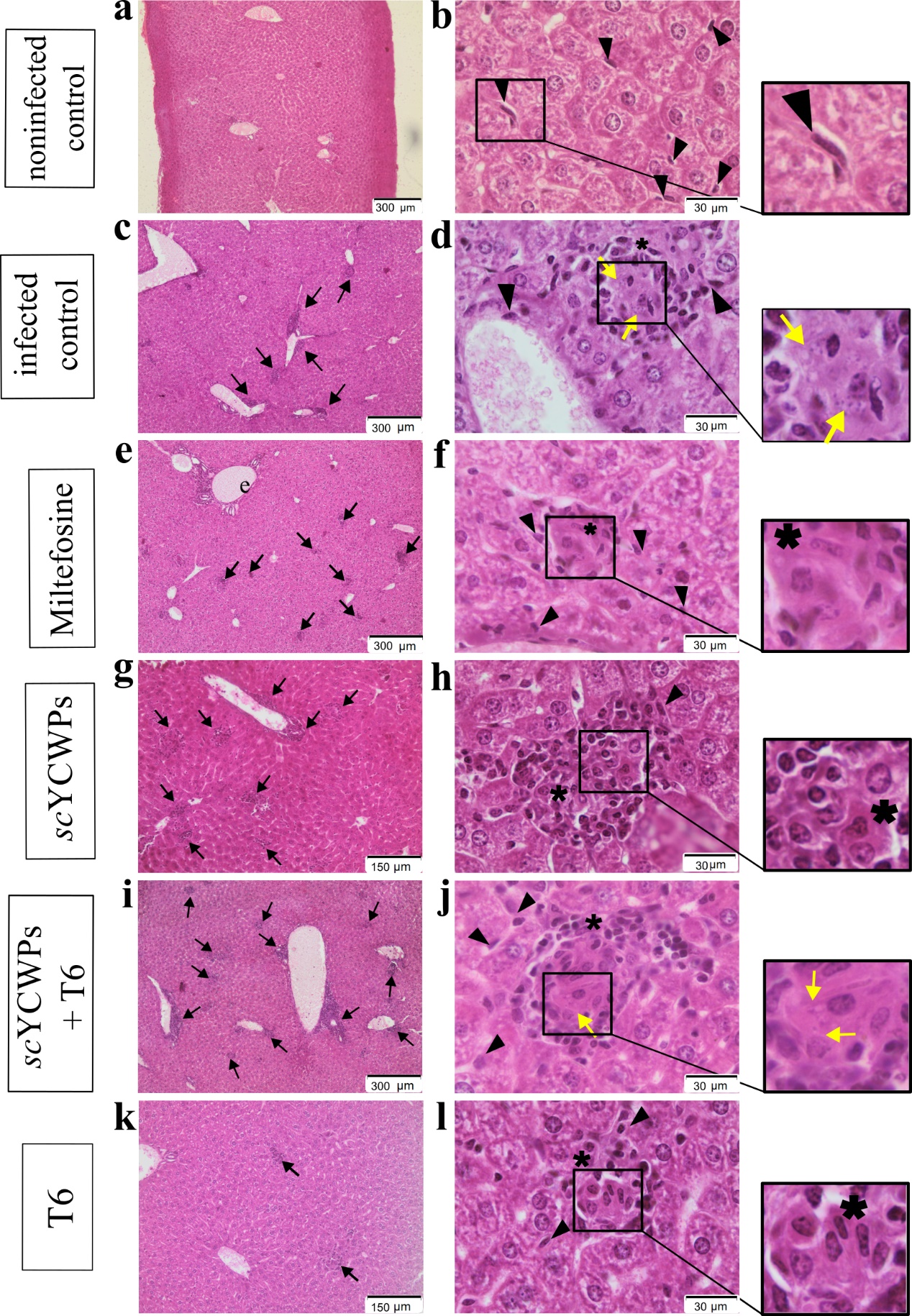

Supplement: Supplementary file 1 — Supplementary Information [file 41598_2019_56647_MOESM1_ESM.docx]
